# Uterine Adenomyosis: From Disease Pathogenesis to a New Medical Approach Using GnRH Antagonists

**DOI:** 10.3390/ijerph18199941

**Published:** 2021-09-22

**Authors:** Jacques Donnez, Christina Anna Stratopoulou, Marie-Madeleine Dolmans

**Affiliations:** 1 Société de Recherche Pour l’Infertilité, 1150 Brussels, Belgium; 2 Université Catholique de Louvain, 1200 Brussels, Belgium; 3 Pôle de Recherche en Gynécologie, Institut de Recherche Expérimentale et Clinique, Université Catholique de Louvain, 1200 Brussels, Belgium; christina.stratopoulou@uclouvain.be (C.A.S.); marie-madeleine.dolmans@uclouvain.be (M.-M.D.); 4 Gynecology Department, Cliniques Universitaires Saint-Luc, 1200 Brussels, Belgium

**Keywords:** adenomyosis, pathogenesis, estrogen, progesterone resistance, medical treatment, GnRH antagonist, linzagolix

## Abstract

Uterine adenomyosis is a common chronic disorder frequently encountered in reproductive-age women, causing heavy menstrual bleeding, intense pelvic pain, and infertility. Despite its high prevalence, its etiopathogenesis is not yet fully understood, so there are currently no specific drugs to treat the disease. A number of dysregulated mechanisms are believed to contribute to adenomyosis development and symptoms, including sex steroid signaling, endometrial proliferation and invasiveness, and aberrant immune response. Abnormal sex steroid signaling, particularly hyperestrogenism and subsequent progesterone resistance, are known to play a pivotal role in its pathogenesis, which is why various antiestrogenic agents have been used to manage adenomyosis-related symptoms. Among them, gonadotropin-releasing hormone (GnRH) antagonists are swiftly gaining ground, with recent studies reporting efficient lesion regression and symptom alleviation. The aim of the present review is to compile available information on the pathogenesis of adenomyosis, explore the etiology and mechanisms of hyperestrogenism, and discuss the potential of antiestrogenic therapies for treating the disease and improving patient quality of life.

## 1. Introduction

Uterine adenomyosis is a commonly encountered chronic condition, estimated to affect approximately 20% of gynecology patients [1,2]. From a histological perspective, adenomyosis is characterized by the presence of endometrium-like tissue inside the myometrium, which it is believed to invade, eventually causing an asymmetrically enlarged uterus [3]. In terms of diagnosis, magnetic resonance imaging (MRI) and transvaginal ultrasound (TVUS) are the techniques of choice, while the presence of lesions is usually confirmed histologically when a surgical specimen is available [4,5]. Based on imaging and histological data, adenomyosis usually presents as a diffuse phenotype involving a number of small lesions dispersed throughout the myometrium, but sometimes it appears in the form of larger nodules (focal adenomyosis) or cysts (cystic adenomyomas) [5,6,7,8].

Manifestations of adenomyosis and their severity are heterogeneous among patients, but usually include heavy menstrual bleeding, chronic pelvic pain, and infertility [2,9]. Increased risk of miscarriage and pregnancy complications also seem to be related to adenomyosis, as demonstrated by a recent meta-analysis [10]. Besides reproduction-associated symptoms, a recently published review on endometriosis compellingly argued the potential systemic effects of this pelvic disease, ranging from cardiovascular conditions to anxiety and depression, in an attempt to further raise awareness of these disorders [11].

Given the high prevalence and severe symptoms of adenomyosis, one can well imagine the heavy socioeconomic burden. Indeed, a population-based study in the United States revealed that an astonishing 82% of adenomyosis patients ended up undergoing a hysterectomy, while 37.6% resorted to chronic use of pain medication [12]. These data highlight the key importance of finding an efficient treatment for adenomyosis-related symptoms and enhancing the quality of life of all these women.

As yet, there is no ‘adenomyosis drug’, but numerous off-label medications have been used over the years, as detailed by Vannuccini and colleagues [13]. Unfortunately, most current therapies either show limited efficacy or have been linked to substantial side effects, prompting continued investigations into novel options. The present review focuses on the pathogenesis of uterine adenomyosis, the diverse role of estrogen in disease development and symptoms, and the potential of emerging treatments using gonadotropin-releasing hormone (GnRH) antagonist against adenomyosis-related symptoms.

## 2. Hypotheses on the Origin of Uterine Adenomyosis

**Figure 1:**
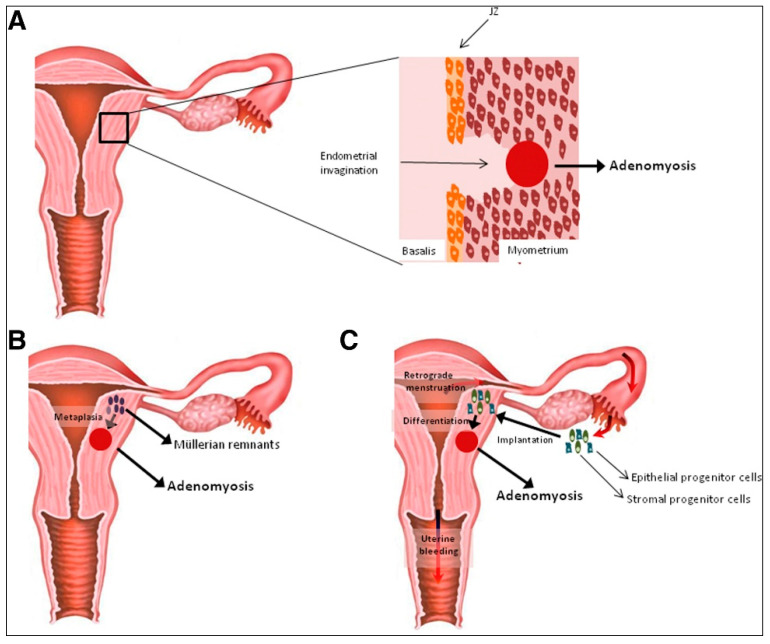
Hypotheses on the origin of uterine adenomyosis. (**A**) Invasion of the myometrium by endometrial tissue upon disruption of the JZ. (**B**,**C**) De novo generation of adenomyotic lesions as a result of (**B**) metaplasia of misplaced embryonic pluripotent remnants or (**C**) retrograde menstruation and subsequent implantation of endometrial progenitor cells in myometrial locations (reprinted with permission from [9]).

Despite being a notoriously enigmatic disease, our understanding of the pathogenesis of adenomyosis has greatly progressed over recent years. To date, there are two main hypotheses explaining its origin: (i) invasion of the myometrium by endometrial tissue via a traumatized endometrial–myometrial junctional zone (JZ); and (ii) de novo generation of endometrial tissue in ectopic locations as a result of either metaplasia of embryonic Müllerian remnants or differentiation of local adult stem cells [2,9,14,15] (Figure 1).

### 2.1. Theory of Endometrial Invasion in the Pathogenesis of Adenomyosis

According to the first and most widely accepted theory originally proposed to shed light on the development of both adenomyosis and endometriosis, basal endometrial tissue invades the myometrium via trauma-inflicted discontinuity of the JZ [15]. In this scenario, locally produced estrogen, combined with that of ovarian origin, creates a hyperestrogenic environment in the uterus, increasing mechanical strain and hence contractions, thereby traumatizing the JZ [15]. Endometrial tissue then escapes the JZ and invades the myometrium, where it establishes itself as an adenomyotic lesion.

This invasive capacity of endometrial cells has been attributed to the process of epithelial to mesenchymal transition (EMT), a phenomenon characterized by loss of cell polarity, destabilization of tight intercellular junctions, and, ultimately, transition into motile mesenchymal cells [16,17]. This process is pivotal to both normal and abnormal wound-healing responses and is therefore consistent with the theory of tissue injury and repair and subsequent invasion [17]. Further studies indeed corroborated the hypothesis of invasiveness, as adenomyotic glands appear to resemble those of eutopic endometrium and most likely originate from them [18]. Moreover, single-cell transcriptomic data detected a clear upturn in genes related to cell motility and cancer-like features in adenomyosis [19]. It has also been hypothesized that estrogen itself drives EMT in adenomyosis, although other studies have proposed inflammation-associated factors as mediators of this process [16,20,21].

### 2.2. Hypothesis of De Novo Generation of Adenomyotic Lesions

An alternative theory on the origin of adenomyosis maintains that ectopic lesions are generated de novo rather than deriving from eutopic endometrium [22]. One possible explanation for this involves the differentiation of misplaced embryonic Müllerian remnants into endometrium-like tissue [22]. This theory is mostly supported by literature reports of organoid structures of Müllerian origin resembling primitive endometrial tissue in normal organs of fetuses, including the posterior uterine wall [23]. According to Batt and Yeh, this tissue may later differentiate into endometrium-like tissue and grow as an ectopic lesion, but this has not yet been experimentally proved [22]. Although not as popular and far less studied than the invasion hypothesis, the concept of Müllerianosis in adenomyosis development may explain some uncommon adenomyosis diagnoses in patients lacking a functional endometrium.

It is now well known that adult stem and progenitor cells reside in the endometrium and menstrual blood [14,24]. They are responsible for physiological endometrial regeneration upon cessation of menstruation, by recreating lost epithelium and vasculature. According to the most popular notion on the pathogenesis of endometriosis, namely Sampson’s theory, viable endometrial fragments are transported via retrograde menstruation and form ectopic lesions by adhering to the peritoneum and proliferating into islets of endometrial tissue [25]. However, only a small number of women with retrograde menstruation go on to develop endometriosis, suggesting the existence of at least one additional determining factor. Endometrial stem cells (ESCs) have been suspected of triggering endometriosis when they are carried and adhere to ectopic locations thanks to their ability to differentiate into different types of cell populations making up the endometrium [14,24]. ESCs may well implant in ectopic uterine locations upon transportation in menstrual blood, establishing adenomyotic lesions in a similar manner. Thus, the missing determinant leading to endometriosis or adenomyosis development could lie in the different numbers and cell capacities of ESCs that facilitate their implantation and propagation [14,26]. Alternatively, fragments of endometrial basalis, which are more commonly found in the menstrual blood of endometriosis patients than disease-free subjects, may contain all the necessary progenitor cells to generate ectopic lesions upon acquiring access to the peritoneum via retrograde menstruation [27].

## 3. Role and Causes of Hyperestrogenism in the Pathogenesis of Adenomyosis

### 3.1. Impact of Estrogen on Endometrial Cells

**Figure 2:**
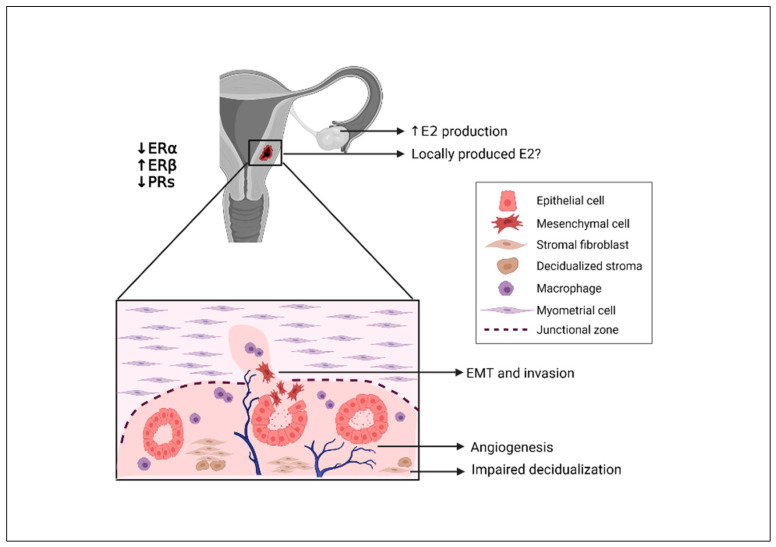
Effects of estrogen during adenomyosis development. Increased ovary-secreted estrogen, potentially combined with that of endometrial origin, triggers an inflammatory response in the endometrium, characterized by macrophage infiltration, angiogenesis, and EMT with subsequent invasion of the myometrium by endometrial cells. At the same time, dominance of ERβ over ERα downregulates PR-B expression, resulting in progesterone resistance and inability of the endometrium to transform into a secretory decidualized state.

Adenomyosis, like endometriosis, is generally regarded to be an estrogen-dependent disease, since a whole range of pathogenic mechanisms rely on its upregulation (Figure 2). It is widely known that estrogen exerts a proliferative effect on the endometrium, while adenomyosis has been repeatedly associated with endometrial cell overproliferation [28]. Indeed, a recent study demonstrated that supplementing culture of endometrial stromal cells from adenomyosis patients with estradiol (E2) significantly boosted their proliferation rates [29]. In addition to proliferation, estrogen has been shown to induce EMT in adenomyosis, a phenomenon frequently blamed for endometrial invasiveness [16,30]. Although both endometrial epithelial and stromal cells are considered invasive in vitro, their invasion capacity appears to increase with the administration of E2 to culture [16,31].

Furthermore, it has been suggested that E2 promotes vascular endothelial growth factor (VEGF) expression in both endometrial epithelial and endothelial cell lines and greater migration capacity of endothelial cells in vitro, whereas blocking E2 attenuates these effects [32]. In subsequent in vivo experiments, E2 treatment was shown to be essential to peritoneal lesion adhesion and vascularization in a mouse model, leading the authors to speculate that this type of interaction is also crucial during human adenomyosis development [32].

### 3.2. Evidence of Hyperestrogenism in the Myometrium

The myometrium also seems to be vulnerable to nonphysiological changes in local estrogen expression and signaling. An imbalance in the estrogen receptor alpha (ERα)/estrogen receptor beta (ERβ) ratio has been reported in myometrial noradrenergic nerve fibers, where a switch to ERα was noted in adenomyosis patients, along with a cycle-independent reduction in the number of nerve fibers [33]. Based on these findings, the authors suggested that estrogen signaling is abnormal in adenomyotic uteri, affecting and possibly disrupting local innervation. Moreover, a recent study found that, in healthy myometrium, expression of G protein-coupled estrogen receptor (GPER) (a transmembrane receptor of estrogen with reduced affinity) cyclically decreased in the secretory compared with the proliferative phase, but this variation was not maintained in adenomyotic myometrium, where expression was constantly higher than in healthy tissue [34].

### 3.3. Potential Interaction of Estrogen and the Immune Response

The numbers, types, activation status and specific roles of immune cells in the endometrium, and especially the functions, differ according to the phase of the menstrual cycle, as they are dependent on local hormone levels [35]. It has been postulated that estrogen and progesterone signaling act synergistically with the immune response to promote disease development and progression, with dysregulation of hormone levels resulting in aberrant immune cell accumulation and activity [36]. Indeed, macrophages and uterine natural killer cells (uNKs), key mediators of innate immunity, have both been reported to be increased in endometrium from adenomyosis patients, particularly in more severe forms of the disease [36,37]. Regarding the adaptive immune system, abnormalities in numbers and the activation status of T lymphocytes have been identified in the endometrium from adenomyosis patients [38,39].

A specific interaction with estrogen has been observed in the case of macrophages, which are thought to participate markedly in lesion progression, innervation, and subsequent pain symptoms [20,40,41]. According to the invasion theory, hyperestrogenism initially traumatizes the JZ, and inflammatory cells, such as macrophages, accumulate in an attempt to repair the damage, eventually leading to chronic inflammation and more estrogen production [15]. Macrophages physiologically express ERs, but their expression appears to be upregulated in endometriosis-derived macrophages, suggesting an interplay between these cells and estrogen [42,43]. To this end, high numbers of macrophages thought to contribute to adenomyosis development may actually be the result of local hyperestrogenism attracting these cells.

### 3.4. Origin of Aberrant Estrogen Signaling in Adenomyosis

The exact mechanisms governing hyperestrogenism in adenomyosis still need to be elucidated, but genetic predisposition is suspected. One study identified differential alleles in key genes involved in estrogen metabolism in women with adenomyosis compared with the control group [44]. Aberrant expression of ERs may also be the underlying cause of dysregulated estrogen signaling in the endometrium from adenomyosis subjects, as evidenced by transcriptome analysis [45]. Indeed, a switch of the ERα/ERβ ratio towards ERβ is considered crucial to endometriosis-related overproliferation, apoptosis inhibition, progesterone resistance, and pain symptoms, as recently reviewed [11,46]. It was also proposed that endometriotic and adenomyotic tissue may biosynthesize estrogen in situ via production of aromatase, but subsequent studies refuted the theory of local aromatase production in endometriosis [47,48,49].

## 4. Evidence of Endometrial Progesterone Resistance

### 4.1. Origin of Progesterone Resistance and the Role of ERs

In the uterus, the role of progesterone signaling is pivotal, ranging from the regulation of uterine contractions and uterotubal transport of sperm, to the establishment of a receptive endometrium for embryo implantation [50]. Endometrial progesterone resistance, a phenomenon frequently associated with aberrant estrogen signaling, has been linked to diseases of the reproductive system, such asadenomyosis, endometriosis, and polycystic ovary syndrome [51,52]. The precise mechanisms impairing progesterone signaling are not fully elucidated, but a chronic hyperestrogenic and inflammatory environment and subsequent epigenetic changes are thought to contribute to an insufficient progesterone response [50]. It is also believed that overexpression of ERβ in ectopic lesions downregulates expression of ERα, thereby hampering ERα-mediated induction of progesterone receptors (PRs) [46,53,54]. Indeed, back in 1997, one study found that PR-A and PR-B did not follow physiological cyclic variation patterns in an ectopic endometrium, potentially indicating the presence of biologically inactive receptors [51]. It was later suggested that PR-B might be entirely absent from endometriotic lesions and even from eutopic endometrium from endometriosis patients in some cases [55]. Consistent with these findings, PR-B expression has been reported to be lower in both eutopic and ectopic endometrium in adenomyosis, especially in the most severe cases [56,57,58]. Insufficient progesterone signaling then downregulates expression of 17β-hydroxysteroid dehydrogenase type 2, an essential enzyme for oxidization of E2, into less active estrone and conversion of hydroxyprogesterone into its active form, further exacerbating local hyperestrogenism and progesterone resistance [53,59].

A link between KRAS gene mutations and low PR expression has also been postulated, further corroborating the notion of estrogenic action inhibiting progesterone signaling in adenomyosis [60]. KRAS is indeed often mutated in endometrial cancer and thought to interact with estrogen signaling pathways. It has also been implicated in the pathogenesis of endometriosis, where gene mutations are present, and its overactivation may lead to progesterone resistance [61,62].

### 4.2. Is Progesterone Resistance Linked to Infertility?

Progesterone is considered the ‘pregnancy hormone’ because of its role in inducing expression of major implantation-related factors in the endometrium, but its dysregulation interferes with the embryo’s capacity to implant (for an in-depth review, see [63]). Decidualization, a series of morphological and functional changes that the endometrium needs to undergo to ensure a receptive environment for the embryo, is dependent on cyclic estrogen and progesterone signaling [50,64]. Disruption of progesterone and its downstream signaling cascades impedes this strictly regulated series of events and may result in embryo implantation failure [63,65]. Although a direct relationship between progesterone resistance and infertility has not yet been established in adenomyosis, endometrial cell decidualization has been found to be impaired, suggesting an inability to respond to progesterone and potentially explaining the frequently reported implantation failures seen in these patients [10,66,67].

## 5. Medical Treatment of Adenomyosis

### 5.1. Current Medical Therapies for Adenomyosis: The Need for Novel Options

Given the high prevalence, debilitating symptoms, and chronic nature of adenomyosis, the need for nonsurgical treatment of the disease is becoming ever more pressing, especially for younger patients. The main objective of treating uterine adenomyosis is symptom management, but the choice of how depends on the woman’s age, reproductive status, and clinical symptoms. Treatment options for women are limited at present and involve use of analgesics or off-label hormone therapies. There is very little specific information available about medical therapy and, to date, no drug has been approved for treatment of adenomyosis [13,68]. Conservative surgery remains a source of controversy and, while some clinical studies into surgical treatment have reported good results in experienced hands [69], the risk of uterine rupture during a subsequent pregnancy is not negligible. Indeed, robust evidence supporting a conservative surgical approach is still lacking.

Progestins may be considered an option as they have, in theory, antiproliferative and anti-inflammatory effects, but progesterone resistance limits their efficacy [13,51,54,68,70]. As previously stated, progesterone resistance in an adenomyotic endometrium and stroma is typical of adenomyosis, similar to observations in deep endometriotic nodules that are commonly associated with uterine adenomyosis [2,5,7,57,70]. Alleviation of both pain and bleeding were reported in a long-term study with dienogest [71], but not confirmed in cases of severe adenomyosis. The levonorgestrel-releasing intrauterine system (LNG-IUS) shows reasonable efficacy, but only if adenomyosis is limited and close to the uterine cavity [13,68,72]. These options are not effective for moderate or severe (full-thickness) disease.

New medications, such as selective progesterone receptor modulators (SPRMs), have also proved ineffective, since SPRMs induce reversible and benign endometrial changes known as progesterone receptor modulator-associated endometrial changes (PAECs) in intramyometrial endometrium [54]. Indeed, Donnez and Donnez reported more severe adenomyotic lesions after ulipristal acetate (UPA) therapy, with greater numbers and severity of cystic adenomyotic lesions [73]. Conway et al. reported the worsening of several ultrasound characteristics of adenomyosis, concomitant with the aggravation of symptoms in UPA-treated adenomyosis patients [74].

As adenomyosis is essentially estrogen-dependent, hormone therapies reducing mitigating estrogens may prevent intramyometrial growth of endometrial glands. GnRH agonists were therefore proposed to both tackle adenomyosis-related hyperestrogenism and decrease proliferative activity in ectopic lesions [75]. However, although GnRH agonists have long been recognized for their efficiency in reducing uterine volume and providing symptom relief, their use remains limited and short term due to their adverse side effects and, importantly, rapid disease recurrence has been observed upon treatment cessation [13,76,77,78]. According to Vannuccini and Petraglia [13,72] and Cope et al. [68], use of GnRH agonists for the management of adenomyosis-related pain and bleeding should only be considered for short-term administration because of their menopausal effects, initial flare-up effect, and slow reversibility. One study did nevertheless report a higher pregnancy rate in adenomyosis subjects undergoing frozen embryo transfer after GnRH agonist pretreatment [79].

### 5.2. Treating Adenomyosis Symptoms with GnRH Antagonists: A Promising New Approach

There is clearly a large unmet need for improved long-term medical therapies for adenomyosis [13]. Barbieri’s estrogen threshold hypothesis suggests managing estrogen levels to minimize side effects while maintaining efficacy in terms of mitigation of symptoms, which could constitute a viable treatment option [54,80].

**Figure 3:**
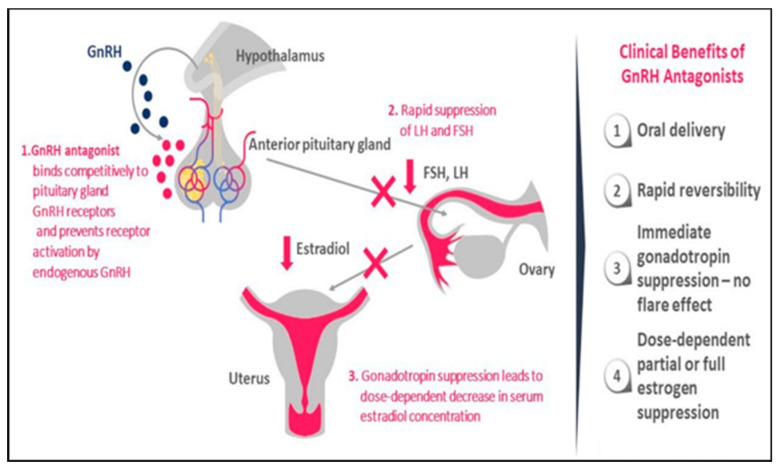
Mode of action and advantages of GnRH antagonist use in clinical practice (reprinted from [54]).

GnRH antagonists have indeed emerged as a potential alternative to allow dose-dependent control of E2 levels [81,82]. As well as their unique capacity to modulate E2 suppression, another advantage of orally active GnRH antagonist over GnRH agonist depot formulations is the absence of the flare-up effect, hence avoiding initially worsening symptoms and rapid reversibility [81,82]. In theory, they could reduce the occurrence of ectopic endometrial implants in the myometrium, relieve adenomyosis-associated pain, diminish uterine volume, and lower the prevalence of hypoestrogenic side effects by modulating the dosage (Figure 3) [54,81].

**Figure 4:**
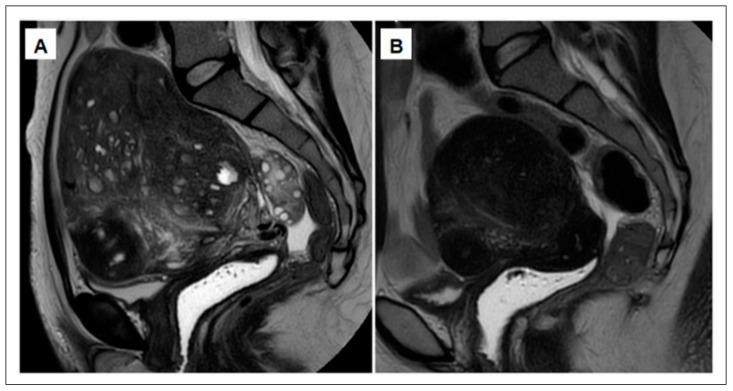
(**A**) MRI showing a very large uterus, consistent with severe full-thickness adenomyosis. (**B**) After a 12-week course of GnRH antagonist (daily dose of 200 mg linzagolix), a significant reduction is observed in both uterine size and adenomyotic foci (adapted from [73]).

Indeed, an interesting case report showed that administration of a GnRH antagonist effectively alleviated symptoms and improved MRI features of adenomyosis [73] (Figure 4). In accordance with this theory, a recent pilot study evaluated the efficacy of a once-daily regimen of 200 mg linzagolix for 12 weeks in women with a confirmed MRI diagnosis of diffuse adenomyosis [4] and adenomyosis-related symptoms [83]. The efficacy endpoint was the change in volume of the adenomyotic uterus from baseline to week 12. Mean ± SD uterine volume was 333 ± 250 cm^3^ at baseline. By 12 weeks, an MRI showed that it had dropped to 159 ± 95 cm^3^, corresponding to a significant (*p* < 0.005) decrease of 55% [83]. There was also a significant reduction in dysmenorrhea and dyspareunia, as well as improvement in quality of life. Serum E2 was fully suppressed during the first 12 weeks and all the women were amenorrheic. Median serum E2 levels were around 12 pg/mL by week 4, which was maintained to week 12. Mild and moderate hot flushes and loss of libido were reported by 25% of women. There was a decrease in bone mineral density, but this could be managed [83].

There is therefore evidence that linzagolix, administered at a high dose for 12 weeks to women with severe symptomatic adenomyosis, substantially reduces uterine volume, decreases uterine bleeding, alleviates pain symptoms, and enhances quality of life. A particular advantage compared with a GnRH agonist is that E2 suppression can be modulated by changing doses (such as switching from 200 to 100 mg) to mitigate hypoestrogenic side effects.

### 5.3. The Potential Link between Adenomyosis and Endometriosis

An important aspect to consider when clinically managing adenomyosis is its potential association with endometriosis and, more specifically, deep endometriotic nodules (DENs). This association is mostly corroborated by their remarkably high rates of coexistence, and applies to both anteriorly and posteriorly located DENs [84,85,86,87,88]. Based on these findings, some authors speculated that adenomyosis and DENs may in fact share a common origin, with DENs being the outcome of adenomyosis or vice versa. In the first scenario, extensive proliferation and progression of adenomyotic lesions may cause them to invade nearby extrauterine tissue, where they form DENs [84,85]. On the other hand, it is possible that regurgitant menstrual flow in the abdominal pelvic cavity, often blamed for endometriosis development, could lead to adenomyosis upon adherence of endometrial cells to the posterior uterine wall [86].

Given this theory and the similar symptoms of adenomyosis and DENs, namely menorrhagia, dysmenorrhea, and infertility, medical treatments for adenomyosis may also be beneficial against endometriosis in patients who suffer from both diseases.

## 6. Conclusions

Uterine adenomyosis is a debilitating condition affecting a considerable number of reproductive-age women worldwide. With recent improvements in our understanding of its pathogenesis and the pivotal role of sex steroid signaling, various drugs have emerged as potential therapeutic agents. Unfortunately, none of them satisfactorily combine efficacy and safety, meaning that most patients eventually undergo a hysterectomy. GnRH antagonists constitute a novel approach, seeking to alleviate hyperestrogenism-related symptoms in adenomyosis subjects. Preliminary data demonstrate encouraging efficacy. More large-scale studies are needed to determine the potential of GnRH antagonists in treating symptomatic adenomyosis patients in routine clinical practice.

## Data Availability

Not applicable.
